# 3D printed system using *ex vivo* porcine eye globes to investigate intracorneal absorption of dexpanthenol from contact lens care solution and eye drops

**DOI:** 10.1016/j.ijpx.2025.100472

**Published:** 2025-12-17

**Authors:** Verena Santer, Adyl-Michaёl El Guamra, Tohru Kawaguchi, Mouad Lamrani, Yogeshvar N. Kalia

**Affiliations:** a Menicon R&D Innovation Centre, Menicon Co., Ltd., Nagoya (Japan), Geneva branch, 1205 Geneva, Switzerland; b PRS Consulting, Nagoya, Japan; c Menicon Co., Ltd., 21-19, Aoi 3, Naka-ku, Nagoya 460-0006, Japan; d School of Pharmaceutical Sciences, University of Geneva, Rue Michel-Servet 1, 1205 Geneva, Switzerland; e Institute of Pharmaceutical Sciences of Western Switzerland, University of Geneva, Rue Michel-Servet 1, 1205 Geneva, Switzerland

**Keywords:** *Ex vivo* ocular model, Simulated lacrimal washout, Dexpanthenol, Contact lenses, Eye drops, 3D printed system

## Abstract

Reduction of the use of animal models in research is encouraged for the sake of animal wellbeing. However, available *in vitro* models in the specific case of topical ocular delivery/penetration studies are often oversimplified by the use of excised corneal or scleral tissue and the frequent lack of dynamic barriers such as the lacrimal outwash. This is why we have developed our novel *ex vivo* porcine eye globe 3D printed laboratory setup with simulated tear flow, using enucleated porcine eyes. This setup was employed to investigate the penetration of a common topical formulation excipient Dexpanthenol (Dxp). First, Dxp deposition in soft contact lenses following usage of SOLOCARE AQUA® care solution was quantified by UHPLC-MS/MS. The subsequent penetration into corneal tissue and aqueous humour of Dxp from SOLOCARE AQUA® care solution treated contact lenses was compared to that following application of eye drop solutions Bepanthen® and Siccaprotect®, containing equivalent Dxp concentrations. The results showed that the Dxp concentration in the anterior segment was three-fold higher after application of the Dxp-containing eye drops as compared to contact lens application. Given that Dxp uptake was greater following the application of the marketed eye drops, this confirmed the safety of the Dxp-containing contact lens care solution. This research demonstrates how topical delivery studies on the ocular surface can be simulated in our novel *ex vivo* porcine eye globe model without the need to sacrifice laboratory animals**.**

## Graphical abstract

**Figure:**
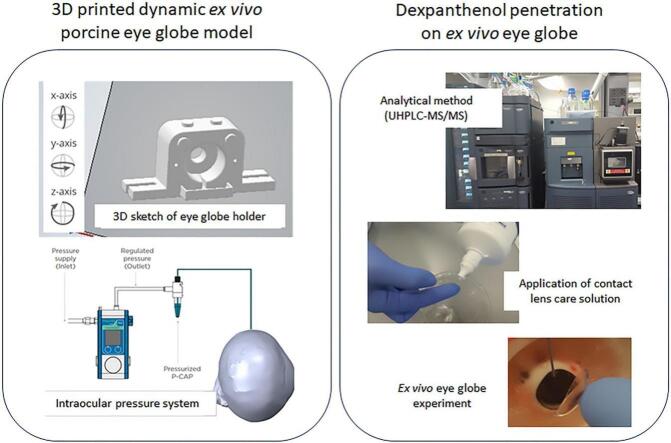

## 1 Introduction

The treatment of ophthalmological diseases is notoriously challenging even though – paradoxically – parts of the organ are exposed to the external environment and easily accessible (Urtti, 2006). The eye globe is divided commonly into 2 sections, the anterior (cornea, aqueous humour and lens) and the posterior (sclera, humour vitreous, retina) segments (Gaudana et al., 2010). These compartments fulfil different roles and have their specificities for ocular drug delivery, possessing diverse target tissues for treatments and barriers to be overcome (Cholkar et al., 2013). Hence, pathologies, as well as the corresponding treatments, are usually distinguished as anterior or posterior segment diseases (Gote et al., 2019).

The posterior segment englobes in its innermost layer the photoreactive cells of the retina processing light stimuli and transmitting the information to the brain through the optic nerve. Diseases affecting the retina such as age-related macular degeneration, diabetic retinopathy and glaucoma are among the leading global causes of blindness with exception of cataract and under-corrected refractive error, which have been defined by the WHO as “avoidable” visual impairment (Steinmetz et al., 2021). The impact of such posterior segment eye diseases resulted in there being more than 19 million vision impairment cases in 2020 (Steinmetz et al., 2021). The treatment is highly challenging due to the isolation of the eye globe from the systemic blood flow by the blood-ocular barrier system and the presence of dynamic and static barriers on the ocular surface and tissues (Urtti, 2006). These barriers significantly reduce the effectiveness of oral or topical drug delivery, leading mostly to the clinical standard praxis of invasive and repeated injections into the vitreous humour (Varela-Fernández et al., 2020; Qi et al., 2023).

In contrast, for anterior segment diseases, topical drug delivery, most commonly involving the administration of eye drops, is usually preferred over systemic administration or injection (Yang and Lockwood, 2022). However, such treatment is far from ideal due to the large loss of material caused by the dynamic barriers such as lacrimal outwash and competing absorption by the conjunctival vessels whose surface is about 17-fold larger than the cornea (Mofidfar et al., 2021). As a result of these different factors, less than 5 % of the actual dose administered is considered to be capable of entering the corneal tissue (Urtti, 2006). Due to those many challenges innovation in drug delivery systems such as mucoadhesive formulations, sustained release, implants *etc.* are of crucial interest in the field, in the search for more effective and longer lasting ocular drug formulations (Grassiri et al., 2021; Maharjan et al., 2019; Ilochonwu et al., 2020; Kompella et al., 2021).

For the study of the pharmacokinetics (absorption) of drugs (and excipients), from novel drug delivery systems or traditional formulations, to the cornea or posterior segment, the gold standard remains the use of *in vivo* animal models, especially rabbits due to their large eyes; however, such studies carry the burden of high cost and ethical considerations on animal wellbeing (Loiseau et al., 2023). The search for more sustainable alternatives is omnipresent in the literature as well as encouraged by the lawmakers in the “3R* principle of “Reduction, Refinement and Replacement” in animal experimentation (Díez-Solinska et al., 2022; Sneddon et al., 2017). In the case of corneal drug penetration *in vitro* studies on cell culture, usually as 2D cell cultures but also 3D models, can be performed; however, they do not accurately reflect the transport-limiting properties of the tissue and mostly provide information on the interaction of the drug with the cells, transporters and receptors and toxicology screening of compounds (Hornof et al., 2005; Elliott and Yuan, 2011; Lieto et al., 2022).

As an alternative to 2D cell models, isolated *ex vivo* cornea – leporine, porcine or bovine – in Franz diffusion cells or Ussing chambers are described, guaranteeing the anatomical barrier functionality of the tissue (*e.g.* epithelial tight junctions) (Shafaie et al., 2016; Pescina et al., 2015; de Hoon et al., 2023; Sebastián-Morelló et al., 2020). Nevertheless, most such setups suffer from the lack of eye lacrimal flow dynamics (Agarwal and Rupenthal, 2016). An “upgraded” version for isolated corneas is achieved by the integration of the excised tissue in a bioreactor that restores the intracorneal pressure and circulation of fluids on the cornea – these systems were mostly developed to enhance corneal endurance for corneal transplants (Trone et al., 2013; Guindolet et al., 2017).

Another type of *ex vivo* model involves the use of entire eye globes, employing mostly animal eyes from slaughterhouse waste, *e.g.* porcine eye globes, due to their physical resemblance in size and structure to the human eye (Menduni et al., 2018; Crespo-Moral et al., 2020). To study the penetration of molecules into the cornea of excised porcine eye globes, static (Santer et al., 2017; Thiel et al., 2002) and dynamic models (Barbalho et al., 2023; Kompella et al., 2006) respectively, without and with simulated tear flow have been developed (Peynshaert et al., 2018). Such models however lacked the possibility to maintain tissue temperature and intraocular pressure, as well as suffering from limited viability due to decay of the tissues.

In order to add to the scientific knowledge of *ex vivo* eye models for pharmacokinetic penetration studies, the aims of this study were (i) to develop our own in-house 3D printed setup for *ex vivo* porcine eye globes equipped with moisturizing and temperature-controlled posterior segment holder, as well as microfluidic vertical simulated lacrimal washout and intraocular pressure modulator, and (ii) to use the *ex vivo* eye globe setup to investigate the adsorption/absorption of Dexpanthenol (Dxp), contained in the SOLOCARE AQUA® contact lens care solution (2 % Dxp) following the recommended cleaning and storage procedures (repeated rubbing, storage overnight) into commercially available soft contact lenses and then onto the porcine cornea, and (iii) to compare the Dxp absorption to that observed from commercially available eye drops: Bepanthen® (2 % Dxp) and Siccaprotect® (3 % Dxp) and to show that the concentrations were below those observed from these marketed products and hence could be considered as safe.

## 2 Materials and methods

### 2.1 Design and fabrication of *ex vivo* porcine eye globe holder

**Fig. 1:**
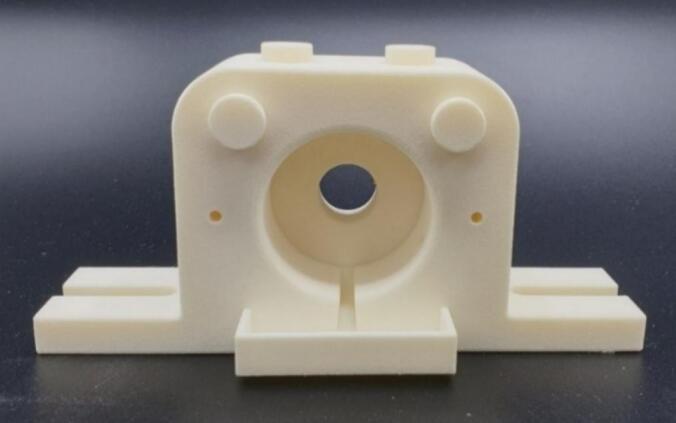
Polyamide 3D printed base structure of *ex vivo* porcine eye globe holder.

For the *ex vivo* porcine eye globe holder setup a polyamide PA12 material 3D printed external housing was designed and manufactured. The mould for the agar gel soft joint and the porcine eye globe holder were designed in SolidWorks 2017. Each part was modelled separately and checked in a simple assembly to confirm the required fit. The STL files were printed by Sculpteo (France) using selective laser sintering (SLS) with Nylon PA12 (white, 60 μm layer thickness) and a standard polished surface finish. The holder included a central cavity to position the eye globe cushioned in agar gel, a liquid collection reservoir on the bottom and upper right and left knobs for the application of the perfusion manifolds for the simulated tear flow as presented in Fig. 1.

**Fig. 2:**
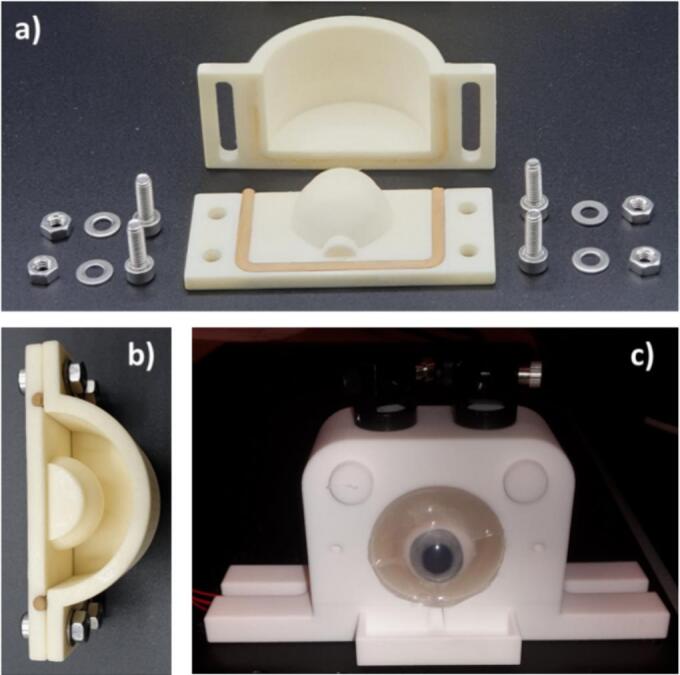
a) Disassembled mould for the preparation of agar gel eye englobing half spheres. b) Assembled mould, ready for the insertion of dissolved agar solution. c) *Ex vivo* porcine eye globe inserted to the setup.

In order to stabilize the excised porcine eye globes in the holder, the posterior segment was cushioned and moistened by 3 % agar gel (Merck, Darmstadt, Germany), which was solidified in custom designed half sphere moulds of different sizes adapted to the porcine eye globe dimensions (Fig. 2). The porcine eye globe was placed onto the lower half-sphere shaped agar gel, once it had solidified. The upper hemisphere was then placed on top and the assembled unit inserted in the designated space on the eye globe holder. It should be noted that the agar gel was slightly soft and not completely rigid; hence its form could be adapted to accommodate variations in the dimensions and heterogeneity of the porcine eyes.

**Fig. 3:**
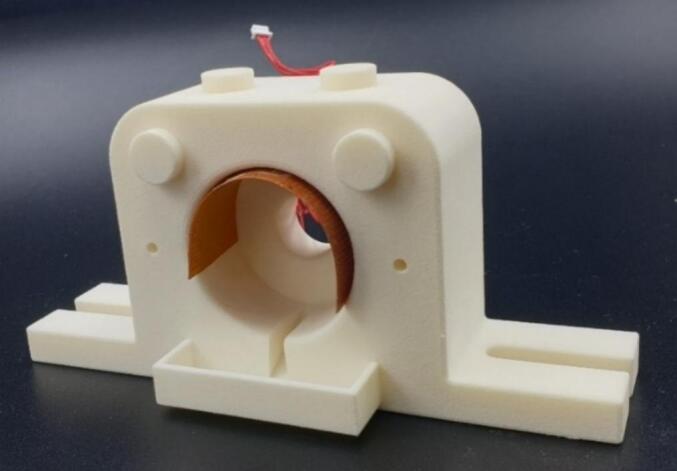
*Ex vivo* porcine eye globe holder 3D printed base structure with flexible resistive foil heater.

Furthermore, a temperature regulation function was added to the system using a TC200 temperature controller and Flexible Resistive Foil Heater both purchased from Thorlabs (Newton, USA). The flexible resistive foil heater regulated by the temperature controller at 37 °C was inserted into the 3D base structure cavity, temperature dissipation over the agar gel and porcine eye was confirmed by a non-contact infrared thermometer (Fig. 3).

**Fig. 4:**
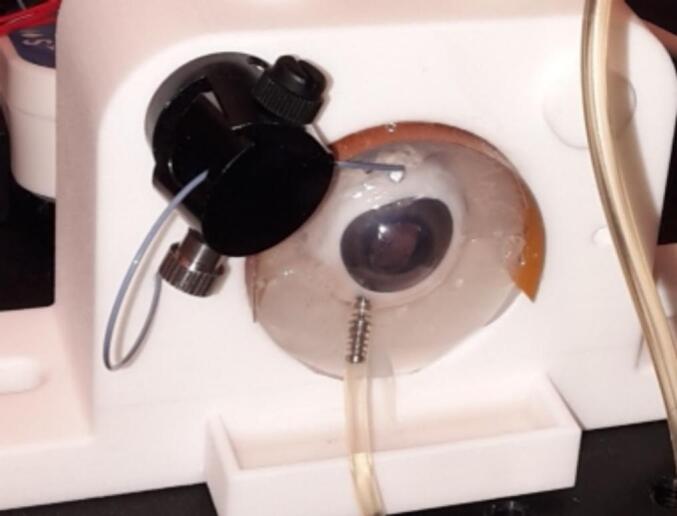
*Ex vivo* porcine eye holder with porcine eye englobed in agar gel with heating system. At the top, the simulated lacrimal flow drop can be seen dispensed on the anterior segment, while on the bottom eye, the pressure injection system is inserted to the vitreous humour and connected to the pressure-based flow controller.

A simulated modulable tear flow was created using microfluidic research equipment composed of LineUp™ LINK, Pressure-Based Flow Controller Flow-EZ™ and Flow unit by Fluigent (Fluigent SA, Le Kremlin-Bicêtre, France) with perfusion manifold on magnetic holder (Biosciencetools, San Diego, USA) (Fig. 4).

As a final functionality of the *ex vivo* eye globe setup, intraocular pressure could be modulated through injection of saline solution by pressure-based flow controller from SMC pneumatics (Yorba Linda, CA) *via* a trocar cannula from Bürki inno med AG, (Widnau, Switzerland) in the posterior segment (Fig. 4) as previously described (Kling et al., 2010; Mattson et al., 2010). The increase in intraocular pression was confirmed using a tonometer iCare IC100 (Icare Finland Oy, Vantaa, Finland).

### 2.2 Analytical method, UHPLC- MS/MS quantification of Dxp

The analytical method for the quantification of Dxp by UHPLC-MS/MS was developed by using a Waters Acquity® UPLC® system (Baden-Dättwil, Switzerland) comprising a binary solvent pump, a sample manager and a Waters XEVO® TQ-S Micro tandem quadrupole detector (Baden-Dättwil, Switzerland). Dxp standard and SOLOCARE AQUA® contact lens care solution (2 % Dxp) were provided by Menicon Co. Ltd. (Nagoya, Japan). To avoid any matrix effect, deuterated Dxp-D6 was bought from TLC Pharmaceutical Standards Ltd. (Aurora, Canada) and used as the internal standard; it was added at a concentration of 100 ng/ml to each sample. Mass spectrometric detection of Dxp and Dxp-D6 were performed with electrospray ionization in positive ion mode using multiple reaction monitoring (MRM). The detection settings for both molecules Dxp and Dxp-D6 are presented in Table 1.

**Table 1:** ESI+ MS/MS settings for the detection of Dxp and the internal standard Dxp—D6.

	**Dxp**		**Dxp-D6**
**Nature of parent ion**	(M + H)+		(M + H)+
**Parent ion (*m*/*z*)**	206.01		212.01
**Daughter ion (m/z)**	75.7	101.85	82.02
**Collision energy (V)**	10	12	12
**Cone voltage (V)**	17	17	5
**Capillary voltage (kV)**	3.98	3.98	3.98
**Capillary temperature (°C)**	350	350	350
**Desolvation gas flow (L/h)**	650	650	650
**Cone gas flow (L/h)**	1	1	1
**Collision gas flow (ml/min)**	0.15	0.15	0.15

Gradient separation was performed with a Waters AQUITY UPLC® BEH6Amide, 2.1 × 100 mm column containing particles of size 1.7 μm and regulated at 40 °C. LC-MS grade acetonitrile (ACN) and methanol (MeOH) (HiPerSolv Chromatonorm; Darmstadt, Germany); ULC/MS grade formic acid (FA) from Brunschwig (Basel, Switzerland) as well as Ultrapure water (Millipore Milli-Q Gard 1 Purification Pack resistivity >18 MΩ cm; Zug, Switzerland) were used to prepare all solutions. The mobile phase consisted of (a) ultrapure water +0.1 % FA and (b) ACN + 0.1 % FA. Gradient elution was performed by increasing the phase (a) to 35 % in the first 0.5 min to 1 min and reduced to 5 % in the following 1.2 min until 3.5 min. The flow rate was 0.3 ml/min and the injection volume was 5 μl. Dxp and Dxp D6 retention time was 1.6 min. The UHPLC-MS/MS method was validated as per ICH Q2 (R1) and FDA, bioanalytical method validation guidelines 2018 (*Validation of Analytical Procedures: Text and Methodology Topic Q2 (R1) International Conference on Harmonisation of Technical Requirements for Registration of Pharmaceuticals for Human Use*, 2005; *Bioanalytical Method Validation Guidance for Industry*, 2018).

#### 2.2.1 Extraction of Dxp from corneal tissue

First, the PTFE filters, used for the preparation of Dxp corneal extraction samples in ACN, were validated. Briefly, *n* = 3 samples of 1 ml Dxp and Dxp-D6 in ACN (100 ng/ml, respectively) were filtered through Chromafil Xtra PTFE single use syringe filters (pore size 0.2 μm). The recovery following filtration of Dxp and Dxp-D6 was 98.5 ± 2.3 %, indicating the absence of adsorption of the analyte and internal standard by the filter material.

Then, porcine corneal discs (0.8 cm^2^) were spiked with a known amount of Dxp dissolved in acetone (31 to 3125 ng/cm^2^). The corneal samples were then cut into small pieces and soaked sequentially first in 2.5 ml ACN for 2 h and then, after removal of the solvent, in fresh 2.5 ml of ACN for overnight extraction. Finally, extraction solvents were combined, filtered, 100 ng/ml of internal standard Dxp-D6 was added and analysed by UHPLC-MS/MS.

#### 2.2.2 Extraction of Dxp from soft contact lenses

Four types of soft contact lenses were investigated in this study, Soft MA®, Miru 1 day® and Miru 1 month® soft contact lenses were provided by Menicon Co. Ltd. (Nagoya, Japan) and the daily use contact lenses 1 day Acuvue® by Johnson & Johnson Vision Care (Limerick, Ireland) were obtained from a local optician.

The extraction of Dxp from the different contact lens materials was established by spiking the contact lenses with known amount of Dxp, 2.5 μg, dissolved in acetone. After the evaporation of acetone, the deposited Dxp was extracted in 5 ml of the following extraction solvents: A) ACN single solvent/single application, B) ACN sequential extraction (section 2.2.1) C) ACN:MeOH 80:20 and D) ACN:MeOH 50:50, at room temperature overnight. Preference was given to the extraction solution A as being consistent to the methodology on corneal tissue.

The extraction procedure was validated at different concentrations 25, 100 and 800 μg (each *n* = 3) Dxp, in 5 ml of the respective optimal extraction solvents depending on the contact lens materials. To each extraction solvent the known concentration of 100 ng/ml internal standard Dxp D6 was added beforehand. The extracts of contact lenses were finally analysed by UHPLC-MS/MS and the amounts of Dxp recovered were compared to the amounts applied.

### 2.3 Dxp deposition into soft contact lenses

The ad/absorption of Dxp from the contact lens solution SOLOCARE AQUA®, containing 2 % Dxp, following standard contact lens hygiene care procedure was investigated. In brief 10 s rinsing and overnight storage with/in SOLOCARE AQUA® was tested on the 4 types of soft contact lenses Soft MA® Miru 1 day® and 1 day Acuvue® and Miru 1 month®); detailed experimental protocol is described in Table 2. The experimental conditions were based on the SOLOCARE AQUA® user recommendations provided by Menicon Co. Ltd.

**Table 2:** Summary of the experimental protocol for the treatment of the different contact lens types with SOLOCARE AQUA® care solution, either for 10 s rinsing or overnight storage*.*

**SOLOCARE AQUA® rinsing 10 s**	**SOLOCARE AQUA® storage over night**
Removal from commercial packaging (n = 3, each contact lens group)	
Rinsing of contact lenses for 10 s with SOLOCARE AQUA® care solution	Storage of contact lenses in Microblock® lens case filled with 8 ml of SOLOCARE AQUA® solution over night
	Next day: 10 s of massage of contact lenses 10 s of rinsing with SOLOCARE AQUA®
Removal of SOLOCARE AQUA® care solution excess	
Extraction of Dxp from contact lenses: MA soft® and Miru 1 month®: 5 ml ACN + Dxp-D6 (100 ng/ml) Miru 1 day® and 1 day Acuvue®: 5 ml ACN/MeOH 80:20 + Dxp-D6 (100 ng/ml)	
Analysis by UHPLC-MS/MS	

### 2.4 Dxp penetration from SOLOCARE AQUA® soaked contact lenses and Dxp eye drops in *ex vivo* porcine eye globe model

Considering the Dxp uptake into different contact lenses, the ones showing the highest Dxp loading – namely, 1 day Acuvue® and Miru 1 month® – following overnight storage in SOLOCARE AQUA® were tested for Dxp penetration into *ex vivo* porcine corneas. Dxp penetration from contact lens application was compared to the hourly (5 times) application of Bepanthen® and Siccaprotect® eye drops, containing 2 % and 3 % of Dxp, respectively. Bepanthen® single dose eye drops (0.5 ml, 2 % Dxp) Bayer (Stulln, Germany) and Siccaprotect® eye drops (multidose flask 10 ml, 3 % Dxp) URSAPHARM (Roggwil, Switzerland) were purchased from a local pharmacy.

The *ex vivo* eye globe experiments were performed as follows: fresh porcine eye globes from 6-month old Swiss noble pigs (100–120 kg, sex not recorded) were obtained from local slaughterhouses (Abbatoir de Loëx Sarl; Loëx Switzerland and Abbatoir de Meinier; Meiner, Switzerland). The adhering muscular tissue was carefully removed from the sclera and the entire eye globes were introduced in a custom-made 3D printed eye globe holder described in section 2.1, except for the intraocular pressure function which was not used for this experiment.

The anterior segment was constantly irrigated at 1 ± 0.5 μl/min with distilled water using the above-mentioned microfluidics system to simulate the physiological tear flow; distilled water was used instead of simulated tear fluid to prevent clogging of the system, *e.g.* through crystallization of salts (Mishima et al., 1966). Following the installation of the fresh eye globes, the corneas were exposed either to the contact lenses or eye drops. In addition, a single layer of medical grade gauze with an area of 1.5 cm^2^ was applied on the cornea (or on top of the contact lenses), in order to establish a more homogeneous distribution of the simulated lacrimal fluid over the entire ocular surface. The gauze was not moistened before application; it was moistened *in situ* by the simulated lacrimal fluid released from the microfluidic system. The corresponding experimental protocols for the four tested conditions (*n* = 6) are summarized in Table 3.

**Table 3:** Summary of the experimental protocol followed for the *ex vivo* porcine eye globe experiments.

	**1 day Acuvue ®**	**Miru 1 month®**	**Bepanthen®**	**Siccaprotect®**
**Pre-treatment**	Overnight storage in SOLOCARE AQUA® care solution in Microblock® lens case Next day: 10 s of massage of contact lenses 10 s of rinsing with SOLOCARE AQUA®		–	–
**Eye globe experiment**	Installation of porcine eye globes into 3D printed holders + simulated lacrimal flow			
	Contact lens application for 6 h		Hourly application of eye drops (5 times)	
**End of experiment**	Removal of contact lens for extraction		–	–
	Cleaning of corneal surface and withdrawal of aqueous humour. Excision of 0.8 cm^2^ corneal tissue disc, snap freezing in isopentane cooled by liquid nitrogen (−196 °C)			
	Storage of samples at −20 °C until extraction and analysis			

At the end of the experiment, the contact lenses were removed, the corneal surfaces were cleaned with distilled water, the aqueous humour recovered by aspiration with a syringe (using a 20G needle inserted through the corneal limbus to extract the entire volume), and an area of 0.8 cm^2^ of the cornea was then snap frozen in liquid nitrogen chilled isopentane. All samples obtained by this procedure were stored at −20 °C until further processing and analysis with UHPLC-MS/MS.

### 2.5 Investigation of Dxp concentration in *ex vivo* porcine eye globe anterior segment

Intracorneal Dxp distribution was investigated by using a layer by layer horizontal slicing method (Santer et al., 2017). For this the frozen corneal tissue samples were mounted on a cryotome (Thermo Scientific CryoStar NX70; Walldorf, Germany) sample holder with cryo embedding media. From the epithelium 4 slices of 100 μm each were cut into the corneal stroma. Dxp deposited in the corneal slices was extracted by sequential extraction in ACN (2 × 0.5 ml) and analysed by UHPLC-MS/MS.

The contact lenses were also extracted in 5 ml extraction solvent over night: Miru 1 month® in ACN, and for 1 day Acuvue® in ACN/MeOH 80:20. In addition, the Dxp content in the aqueous humour was also quantified. To do this, the aqueous humour was diluted in 1 ml ACN. To all samples 100 ng/ml Dxp D6 was added and were filtered by PTFE syringe filters.

## 3 Results and discussion

### 3.1 Analytical methods validation

No interference with the components of corneal extracts was detected at the retention time of 1.6 min from Dxp and Dxp-D6 spiked porcine corneal extracts in ACN (Table 4).

**Table 4:** Comparison of the area under the curve and signal to noise ratio of the analytes (Dxp and Dxp D6) compared to blank corneal extract.

	**10 ng/ml Dxp**	**corneal extract**
**Area**	2502.81	68.83
**Signal/noise**	864.99	16.66
	**100 ng/ml Dxp D6**	**corneal extract**
**Area**	22,929.47	12.04
**Signal/noise**	3944.49	2.34

Calibration curves were constructed by plotting the ratio of Dxp and Dxp-D6 peak area (cps/min) against the ratio of Dxp and Dxp D6 nominal concentrations (ng/ml) and a good linear fit was found in the concentration range of 1–700 ng /ml. Correlation coefficients for all calibration curves were superior to 0.99. The lowest amount of Dxp to be detected (LOD) and lowest limit of Dxp to be quantified (LOQ) were found to be 0.7 ng/ml and 2 ng/ml respectively.

Intra- and inter-day precision and accuracy were assessed using 2, 100 and 700 ng/ml Dxp solutions in ACN spiked with corneal extracts (Table 5).

**Table 5:** Intra- and inter-day precision and accuracy for quantification of Dxp (values are given as mean ± SD).

	**Intraday (*n*** **=** **3)**		**Interday (*n*** **=** **9)**			
Theoretical [Dxp] (ng/ml)	Experimental [Dxp] (ng/ml)	RSD (%)	Recovery (%)	Experimental [Dxp] (ng/ml)	RSD (%)	Recovery (%)
2	1.96 ± 0.05	2.8	97.4	1.97 ± 0.06	2.8	98.7
100	98.58 ± 1.31	1.3	98.6	98.64 ± 0.98	1.0	98.6
700	700.81 ± 8.41	1.2	100.1	710.77 ± 5.86	0.8	101.5

The mean recoveries observed for intra-day measurements were from 97.4 to 100.1 % (RSD 1.2–2.8 %) and for inter-day analysis were between 98.7 and 101.5 % (RSD 0.8–2.8 %). The method was considered as accurate and precise as all measured values were within the acceptance limits of the ICH Q2 (R1) and FDA Bioanalytical Method Validation (2018) guidelines (*Validation of Analytical Procedures: Text and Methodology Topic Q2 (R1) International Conference on Harmonisation of Technical Requirements for Registration of Pharmaceuticals for Human Use*, 2005; *Bioanalytical Method Validation Guidance for Industry*, 2018).

#### 3.1.1 Extraction of Dxp from corneal tissue and contact lenses

Reliable extraction procedures, enabling the recovery of all deposited Dxp from corneal tissue and different soft contact lens types were developed. In the case of the corneal tissue, sequential extraction was found more effective than the use of a single extraction solution as had been previously reported (Lapteva et al., 2019). The sequential extraction protocol with pure ACN could be validated with a recovery above 99 % of Dxp for the tested concentration range of 31.5–3125 ng of Dxp.

For the Dxp extraction from the different contact lenses however the same extraction procedure using pure ACN was not successful for all the types of contact lenses investigated in this study as can be seen in Table 6 summarizing the extraction recovery of Dxp.

**Table 6:** Overnight extraction recovery of Dxp from spiked contact lenses in either (A) ACN single solvent, (B) sequential ACN, (C) ACN 80 % and MeOH 20 % and (D) ACN 50 % and MeOH 50 % (*n* = 3). Contact lenses Soft MA® and Miru 1 month® were not investigated for extraction condition C and D due to successful extraction with condition A. The recovery using the final (optimal) method selected is indicated in bold.

	**Extraction conditions (A-D) and Dxp recovery (%)**			
**Soft contact lens**	**A**	**B**	**C**	**D**
Soft MA®	**99.9 ± 3.4**	102.4 ± 1.1	–	–
Miru 1 day ®	47.2 ± 4.6	48.5 ± 7.5	**96.6 ± 3.9**	93.3 ± 8.0
1 day Acuvue®	66.8 ± 14.3	48.1 ± 0.9	**100.8 ± 3.3**	98.7 ± 4.5
Miru 1 month®	**114.4 ± 1.5**	103 ± 4.7	–	–

From the extraction screening it can be seen that depending on the soft contact lens material class, the optimal Dxp extraction solvent differed. For Soft MA® and Miru 1-month® pure ACN was found suitable, whereas for Miru 1 day® and 1 day Acuvue® the addition of 20 % MeOH was needed. The extraction efficiencies of those extraction solutions were further validated by applying 3 different amounts of Dxp (25, 100 and 800 μg) and found above 90 % when respective optimized extraction procedure was followed.

### 3.2 Dxp ad/absorption onto soft contact lenses following use of SOLOCARE AQUA® care solution

Four types of soft contact lenses were investigated in this study, comprising representatives of FDA classification group 1, 2 and 4 hydrogel contact lenses (Soft MA® Miru 1 day® and 1 day Acuvue®, respectively) and silicone hydrogel contact lens (Miru 1 month®) (Giedd, n.d.). The FDA classification of the materials is based on the water content and ionicity of the material, as described in Table 7.

**Table 7:** Overview of tested soft contact lenses, materials and producer.

	**Soft MA®**	**Miru 1** **day®**	**1 day Acuvue®**	**Miru 1 month®**
**Contact lens** **classification**	Group 1 Non-ionic hydrogel H_2_O < 50 %	Group 2 Non-ionic hydrogel H_2_O > 50 %	Group 4 Ionic hydrogel H_2_O > 50 %	Group (5) Silicone hydrogel
**Material**	Govafilcon A	Hioxifilcon D	Ethafilcon A	Asmofilcon A
**Producer**	Menicon	Menicon	Johnson & Johnson	Menicon

A broad spectrum of soft contact lens materials was investigated to determine whether the ad/absorption of Dxp might be related to the contact lens material. Group 3 of the contact lens materials classes was not represented due to its sporadic / abandoned use in the contact lens industry.

**Fig. 5:**
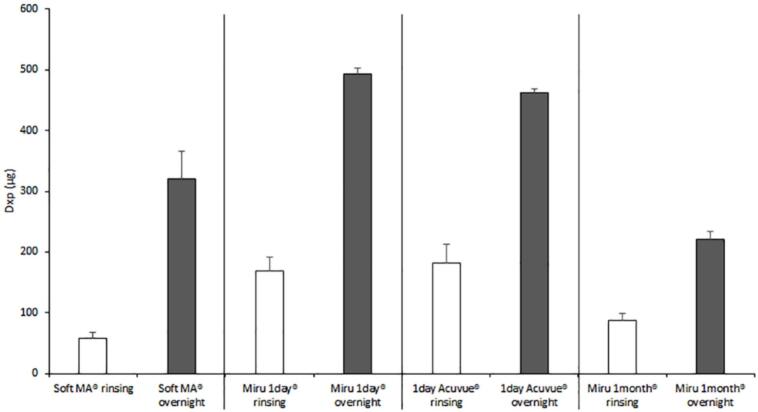
Amount of Dxp extracted from Soft MA®, Miru 1 day®, 1 day Acuvue® and Miru 1 month® following rinsing for 10 s (white columns) or overnight storage (dark grey columns) with SOLOCARE AQUA® care solution (*n* = 3, mean ± SD). (For interpretation of the references to colour in this figure legend, the reader is referred to the web version of this article.)

The deposition of Dxp in the soft contact lenses was quantified by UHPLC-MS/MS following quick rinsing (10 s) or overnight storage with the SOLOCARE AQUA® care solution and subsequent overnight extraction. The results of Dxp contact lens deposition are summarized in Fig. 5.

Exposure of the different contact lenses to SOLOCARE AQUA® solution for either 10 s rinsing or to overnight storage, revealed a clear increase of Dxp deposition in all contact lenses when exposure time was increased. Group 2 and 4 type contact lenses, namely Miru 1 day® and 1 day Acuvue®, had a statistically significant higher Dxp content in both experimental conditions compared to Group 1 and silicone contact lenses, Soft MA® and Miru 1 month®^,^ respectively. This was thought to be linked to the contact lens water content, which was above 50 % for Miru 1 day® and 1 day Acuvue® which is believed to allow higher exchange with SOLOCARE AQUA® care solution compared to SOFT MA® and Miru 1 month® which have water content below 50 %.

### 3.3 Intracorneal penetration of Dxp

Next, the penetration of Dxp into the anterior segment from soft contact lenses, Miru 1 month® and 1 day Acuvue®, soaked overnight with the SOLOCARE AQUA® care solution was investigated. The experiment compared application of the treated soft contact lenses for 6 h with the hourly application of Siccaprotect® and Bepanthen® eye drops (over the same interval) on the *ex vivo* porcine eye globe models cornea undergoing constant 1 μl/min simulated lacrimal flow.

Dxp (D-Panthenol), is the alcoholic precursor of vitamin B5 (pantothenic acid), which itself is a precursor of Coenzyme A (CoA) (Dexpanthenol, n.d.; Leonardi and Jackowski, 2007), an essential cofactor involved in a myriad of biological processes of the cellular metabolism including the biosynthesis of fatty acids which in case of epithelia such as the stratum corneum are crucial to maintain their physiological function (Proksch et al., 2017).

Dxp is a cosmetic ingredient with GRAS status (generally recognized as safe) without any restriction in use (Scott et al., 2022) and is widely added in topical skin formulations as ointment, emulsion, or solution, for its moisturizer and barrier improving effect (Ebner et al., 2002) (Gorski et al., 2020). For the same effect, Dxp is also widely used in ophthalmic products as an excipient in eye drops, gels which often claim an increase in corneal healing, although the clinical evidence is still scarce (Göbbels and Gross, 1996; Sabur and Acar, 2023; Hamdi, 2018; Mencucci et al., 2020). The main reason for the addition of Dxp in such ophthalmological products is its ability to bind water, as is the case for the SOLOCARE AQUA® contact lens care solution in which the combination of Dxp and Sorbitol are covering this function.

**Fig. 6:**
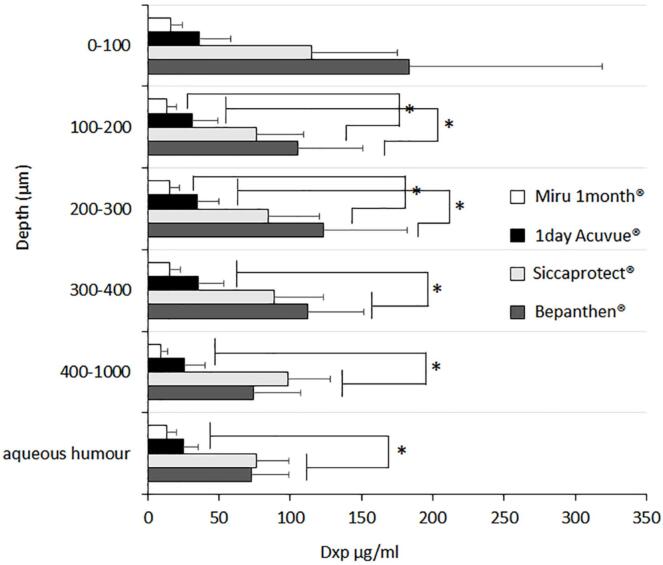
Dxp depth distribution of in corneal slices and the concentration of Dxp in the aqueous humour following the 6 h application of SOLOCARE AQUA® overnight stored soft contact lenses (Miru 1 month® and 1 day Acuvue®) and hourly eye drop instillation (5 times) of Bepanthen® and Siccaprotect®. * indicates statistically significant difference (i. aqueous humour – 3-fold higher deposition with the eye drop group *vs.* the contact lens group (*p* < 0.0014); ii. 400–1000 μm – 3-4 fold higher deposition with eye drops (*p* < 0.0108); iii. 300–400 μm – 3-4 fold higher deposition with eye drops (*p* < 0.0179); iv. 200–300 μm and 100–200 μm – Bepanthan® showed higher deposition than both contact lenses but Siccaprotect® was only superior to Miru 1 month®) (*n* = 6, mean ± SD). (For interpretation of the references to colour in this figure legend, the reader is referred to the web version of this article.)

However, it is important to consider that, as an excipient in a medical device, Dxp penetration into the ocular tissue is not envisioned by the manufacturers, and little is known about the thresholds or concentrations of such. The recovered amounts of Dxp from the corneal slices and aqueous humour are shown in Fig. 6 expressed as concentration of Dxp (μg/ml).

It was possible to note that Dxp deposition following the 6 h application of SOLOCARE AQUA® solution treated contact lenses (Miru 1 month® and 1 day Acuvue®) resulted in a statistically significant lower Dxp deposition in the corneal tissue and aqueous humour than either Bepanthen® or Siccaprotect® eye drops. This result evidenced the higher Dxp delivery to the cornea through direct eye drop instillation as compared to the contact lens application – confirming that transfer of Dxp from the SOLOCARE AQUA® solution treated contact lenses was inferior to that observed by direct application of marketed Dxp products. This also demonstrated the impact of the greater Dxp content in the eye drops on deposition: after overnight treatment of the soft contact lenses, approximately 450–500 μg of Dxp was adsorbed, whereas the application of five drops of Bepanthen® and Siccaprotect® (equivalent to a volume of ∼250 μl) corresponded to the administration of 5 and 7.5 mg of Dxp from the eye drops, respectively.

Comparison of the results obtained with Bepanthen® and Siccaprotect® eye drops revealed that despite the latter's higher Dxp concentration of 3 %, the total concentration of deposited Dxp was not superior to Bepanthen®, despite the greater Dxp content. These findings can be explained by the different compositions of the two eye drop products. Bepanthen® eye drops were found to be more viscous and tissue adhesive due to the presence of 0.15 % of hyaluronic acid in the formulation. Siccaprotect®, on the other hand, contains 1.4 % of polyvinyl alcohol, which is reported to increase the ocular contact time however with inferior effectiveness as compared to hyaluronic acid (Bartlett and Jaanus, 2007). Therefore, it was assumed that the corneal washout of the eye drops was more efficient and quicker for Siccaprotect® compared to Bepanthen®, which could explain the lack of increased deposition of Dxp despite the higher content in the product.

Lastly as can be seen in Fig. 6, an equal distribution of Dxp from the epithelial layer down to the aqueous humour was found for each of the 4 tested experimental conditions. It was assumed that during the 6 h of experiment, Dxp could diffuse from the application site down to the aqueous humour. However, in contrast to classical passive drug permeation studies, given the continuous washout of the contact lenses and eye drops thanks to the simulated tear flow, the concentration gradient from the external to the inner layers was eliminated, so that the deposited Dxp could equilibrate throughout the tissues. To support this hypothesis, the amount of Dxp recovered from the soft contact lenses following the 6 h application was 0.5 to 2 % of the initial amount recovered following overnight storage in SOLOCARE AQUA® care solution.

It also has to be noted that Dxp is a very small hydrophilic molecule (MW: 205 Da and LogP: −1.92, (PubChem Dexpanthenol, n.d.)), hence its diffusion is only limited by the corneal epithelium (approx. 50–90 μm). Whereas the mostly aqueous layers such as stroma and endothelium as well as the aqueous humour do not present any obstacle for its diffusion (Urtti, 2006).

## 4 Conclusions

This study describes the conceptualization and realization of a novel 3D printed *ex vivo* porcine eye globe model with dynamic simulated tear flow, temperature and intracorneal pressure simulation (although this last feature was not used in the present study). Through the model, the relative penetration of Dxp from soft contact lenses treated in Dxp containing SOLOCARE AQUA® care solution and commercially available Dxp containing eye drops (Siccaprotect® and Bepanthen®) into the anterior segment of excised porcine eyes was investigated. Interestingly, it was shown that the direct application of Dxp containing eye drop formulations resulted in increased deposition of Dxp to the anterior segment compared to the SOLOCARE AQUA® stored soft contact lenses. Such a study, avoiding the use of laboratory animals, has a major impact for example in comparative studies between CE marketed medical devices as was the case for SOLOCARE AQUA® and eye drops as Bepanthen® and Siccaprotect®. Through the study, the relative safe use of SOLOCARE AQUA® contact lens care solution in regard of Dxp ocular absorption could be confirmed. A corollary of the findings presented here is that it could be of interest to pursue the development of contact lens based corneal delivery system for Dxp given reports that (i) it was able to reduce corneal neovascularization and inflammation in rats after induction of chemical burns (Isleyen et al., 2025) and (ii) facilitate corneal regeneration (Knorring, 2023).

Therefore, we refer to this *ex vivo* porcine eye globe model as a benchmark system to be used for ocular drug delivery studies using a slaughterhouse waste product with important anatomically similarity to the human organ plus simulated dynamic barrier and organ properties *in vivo*.

## CRediT authorship contribution statement

**Verena Santer:** Writing – review & editing, Writing – original draft, Validation, Methodology, Investigation, Data curation, Conceptualization. **Adyl-Michaёl El Guamra:** Writing – review & editing, Validation, Methodology. **Tohru Kawaguchi:** Validation, Resources, Project administration, Funding acquisition. **Mouad Lamrani:** Validation, Resources, Project administration, Funding acquisition. **Yogeshvar N. Kalia:** Writing – review & editing, Validation, Supervision, Conceptualization.

## Funding

This research received no external funding.

## Declaration of competing interest

The authors declare the following financial interests/personal relationships which may be considered as potential competing interests:

Verena Santer and Adyl-Michaёl El Guamra, were employees of Menicon R&D Innovation Centre, Menicon Co., Ltd. at the time of writing.

Mouad Lamrani is an employee of Menicon Co., Ltd. (Nagoya, Japan).

SOLOCARE AQUA® contact lens care solution (2 % Dxp) is manufactured by Menicon Co. Ltd. (Nagoya, Japan).

## Data Availability

Data will be made available on request.
